# The Use and Effectiveness of Mobile Apps for Depression: Results From a Fully Remote Clinical Trial

**DOI:** 10.2196/jmir.6482

**Published:** 2016-12-20

**Authors:** Patricia A Arean, Kevin A Hallgren, Joshua T Jordan, Adam Gazzaley, David C Atkins, Patrick J Heagerty, Joaquin A Anguera

**Affiliations:** ^1^ Department of Psychiatry and Behavioral Sciences University of Washington Seattle, WA United States; ^2^ Department of Neurology University of California San Francisco San Francisco, CA United States; ^3^ Department of Psychiatry University of California San Francisco San Francisco, CA United States; ^4^ Department of Biostatistics in the School of Public Health University of Washington Seattle, WA United States

**Keywords:** depression, mobile apps, RCT, randomized controlled trial, cognitive training, iPST, problem-solving therapy

## Abstract

**Background:**

Mobile apps for mental health have the potential to overcome access barriers to mental health care, but there is little information on whether patients use the interventions as intended and the impact they have on mental health outcomes.

**Objective:**

The objective of our study was to document and compare use patterns and clinical outcomes across the United States between 3 different self-guided mobile apps for depression.

**Methods:**

Participants were recruited through Web-based advertisements and social media and were randomly assigned to 1 of 3 mood apps. Treatment and assessment were conducted remotely on each participant’s smartphone or tablet with minimal contact with study staff. We enrolled 626 English-speaking adults (≥18 years old) with mild to moderate depression as determined by a 9-item Patient Health Questionnaire (PHQ-9) score ≥5, or if their score on item 10 was ≥2. The apps were (1) Project: EVO, a cognitive training app theorized to mitigate depressive symptoms by improving cognitive control, (2) iPST, an app based on an evidence-based psychotherapy for depression, and (3) Health Tips, a treatment control. Outcomes were scores on the PHQ-9 and the Sheehan Disability Scale. Adherence to treatment was measured as number of times participants opened and used the apps as instructed.

**Results:**

We randomly assigned 211 participants to iPST, 209 to Project: EVO, and 206 to Health Tips. Among the participants, 77.0% (482/626) had a PHQ-9 score >10 (moderately depressed). Among the participants using the 2 active apps, 57.9% (243/420) did not download their assigned intervention app but did not differ demographically from those who did. Differential treatment effects were present in participants with baseline PHQ-9 score >10, with the cognitive training and problem-solving apps resulting in greater effects on mood than the information control app (χ22=6.46, *P*=.04).

**Conclusions:**

Mobile apps for depression appear to have their greatest impact on people with more moderate levels of depression. In particular, an app that is designed to engage cognitive correlates of depression had the strongest effect on depressed mood in this sample. This study suggests that mobile apps reach many people and are useful for more moderate levels of depression.

**ClinicalTrial:**

Clinicaltrials.gov NCT00540865; https://www.clinicaltrials.gov/ct2/show/NCT00540865 (Archived by WebCite at http://www.webcitation.org/6mj8IPqQr)

## Introduction

Major depressive disorder affects approximately 7% of the US population in a given year [1], and approximately 16% of all individuals will experience major depression at least once in their lifetime [2]. Despite the fact that depression is treatable [3,4], less than a quarter of individuals receive adequate care for this illness [1] due to treatment access barriers, such as time and transportation constraints, long waiting lists, and a dearth of trained professionals to provide high-quality care [5-7]. Access problems could be easily mitigated through the use of technology; several studies have already demonstrated that telemedicine and Internet-based approaches are feasible and as effective as in-person treatment [8,9]. The success of these distance approaches has resulted in considerable interest in the use of mobile phone apps as an alternative care delivery platform. Not only do mental health apps have tremendous reach, but also patients can access these tools whenever they feel the need and as often as they like without having to wait until a mental health professional is available [10,11]. Such reach is evident when considering that 68% of all adults in the United States own a smartphone, and approximately 45% own a tablet device [12].

Hundreds of apps for depression are available for download on one’s smart device [13-15], with the majority of these apps designed to be self-guided. While a few proof-of-concept studies and small-scale randomized controlled trials (RCTs) have been conducted, to our knowledge, none have compared theoretically driven interventions versus controls, nor have they investigated the effects of these apps under real-world conditions [16]. Furthermore, we know little about the people who download depression treatment apps in terms of symptom severity, disability, and use of more traditional mental health services. Although a few proof-of-concept studies have found that adherence to depression app guidelines tends to be poor over time [17-21], there is little information about the relative use patterns between different types of apps. We conducted a large-scale RCT (NCT00540865) of 3 different depression apps to answer the following questions: (1) Who downloads mobile apps for depression? (2) How do people who download these apps use them? (3) What is the impact of these apps as they are typically used? (4) What are the methodological issues inherent in conducting a fully remote RCT? We have already published the data on the methodological issues in the recruitment and retention of participants into a large-scale, remote RCT [10]. We report here on the characteristics of people who download and use depression apps, what their use patterns are like, and how effective these interventions are in light of typical use patterns typically deployed in the community.

## Methods

### Study Design

This was a fully remote, randomized clinical field trial comparing 2 active apps and a control app for mood [10], and to test the feasibility of remote research using mobile phone apps. We recruited participants through a variety of Web-based advertising sources, including Craigslist (Craigslist Inc, San Francisco, CA, USA), Google AdWords (Google Inc, Mountain View, CA, USA), and social media outlets (eg, Twitter; Twitter, Inc, San Francisco, CA, USA) from all 50 US states. All potential participants learned about the study through a website explaining study details, which led to a screening protocol using automated software (SurveyGizmo; Widgix, LLC, Boulder, CO, USA) to determine eligibility. Informed consent was conducted through a 2-minute video explaining the study risks and benefits, and the payment structure over the course of the study. In addition to a PDF file of the consent form was provided. Participants had to complete a 3-item quiz testing their understanding of the study to advance to the randomization phase.

Participant contact was minimal. Study staff contacted participants via email or short message service (SMS) text messaging to remind them to use their intervention or assessment app if they had 3 consecutive days of missing data. Aside from this, participants were contacted only when they (1) were due with a payment, or (2) reached out to study staff for technical support. Contact was primarily through email or SMS, with occasional phone call meetings if needed to help participants download their intervention apps. Ethical approval for the trial was granted by the University of California, San Francisco, Committee for Human Research.

### Participants

To be eligible, participants had to be English speakers, be at least 18 years old, and own a smartphone (iPhone or an Android device) with Wi-Fi or third- or fourth-generation capabilities. Because 1 of the 2 interventions was available only on the iOS mobile operating system and had to be used on devices with a visual field larger than that on a typical smartphone, participants had to own an Apple iPad 2.0 or newer version.

Participants had to obtain a score of 5 or more on the 9-item Patient Health Questionnaire (PHQ-9 [22]) or a score of 2 or greater on item 10 of the PHQ-9 (“If you checked off any problems, how difficult have these problems made it for you to do your work, take care of things at home, or get along with other people?”). The decision to include participants with mild symptoms of depression, rather than limit the sample to those with moderately severe depression (eg, PHQ-9 score >10), was based on our intent to test the use and effects of these apps in people with a range symptoms of depression, as well as to determine what proportion of participants who downloaded depression apps fell into mild, moderate, or severe ranges of depression. The only exception to this rule was suicidal ideation. Participants with a PHQ-9 suicide item score of 1 or more were referred to the suicide hotline. We randomly assigned participants to 1 of the 3 apps using a random number generator built into the eligibility survey.

### Baseline Assessments

We collected information on demographics, depression severity using the PHQ-9 [22], functional disability using the Sheehan Disability Scale (SDS [23]), anxiety using the Generalized Anxiety Disorder 7-item scale (GAD-7 [24]), history of mania or psychosis using the Improving Mood-Promoting Access to Collaborative Treatment (IMPACT) assessment of mania and psychosis [25], and alcohol use using the Alcohol Use Disorders Identification Test (AUDIT-C) [26]. We also collected data on self-reported quality of sleep, current use of mobile phone apps, and engagement in outside mental health treatment. Participants in all groups were sent these surveys and were paid US $15.00 for completing the baseline assessment. All treatments and assessments were delivered over the participants’ smart devices. Human interaction was limited to reminders sent via SMS or email based on each participant’s stated preferences.

### Procedures for App Access

Once participants completed the consent process, a secure, 1-user valid link to a secure webpage was sent to participants’ email addresses that contained a brief personalized YouTube video explaining how to download and then use their assigned intervention. This webpage also contained a link to automatically download said apps to the participants’ phone or iPad.

### Intervention Apps

#### Cognitive Control App (Project: EVO)

Participants randomly assigned to this condition were encouraged to use Project: EVO (Akili Interactive Labs, Larkspur, CA, USA) 6 times a week for approximately 30 minutes per day. This app is designed as a video game that modulates cognitive control abilities, a common neurological deficit seen in depression [27]. The app uses adaptive algorithms to adjust the intervention’s difficulty to the user’s level of proficiency over time. Previous work that Project: EVO was derived from demonstrated at this dosage that its prescribed use could improve cognitive control in older adults [28], with preliminary evidence for a similar effect on depression [29].

#### Problem-Solving Therapy App (iPST)

Participants were to use the problem-solving app iPST as often as possible each week, with a minimum of once per week being the typical amount undertaken in a clinical setting. iPST is based on problem-solving therapy, which focuses on a 7-step model to manage mood. In this app, participants choose a goal and are guided through a 7-step process to create an action plan. Problem-solving therapy is an evidence-based treatment [30] and is particularly effective in treating depression [3,31].

#### Information Control (Health Tips)

Participants in this condition were given an app that provided daily health tips for improved health, such as self-care (eg, taking a shower) or physical activity (eg, taking a walk). Although it provided daily advice on improving one’s health, it is not tied to any specific theory, similar to supportive control treatments. Participants were not required to act on the health tip.

### App Design

All 3 apps were developed by professionals with user-centered design method experience (Project: EVO, Akili Interactive Labs, Larkspur, CA, USA; iPST, Wow Internet Labz Pvt Ltd, Bengaluru, India; and Health Tips, Ginger.io, San Francisco, CA, USA) to maximize engagement and minimize app use burden, a common problem associated with app adherence [32,33]. Participants were expected to use their assigned app as instructed for 1 month. The survey app had internally programmed reminders to notify the user that a new assessment was ready for completion, or that they had not completed a given assessment 8 hours after it was originally transmitted. For each intervention app, our team of research assistants monitored participants’ use of their assigned apps using a custom Web-based dashboard. If a participant had not used Project: EVO or iPST in 72 hours, they were sent an email or SMS (based on their indicated preference) reminding them to use their assigned app. If participants did not use their app within the next 72 hours, no further reminders were sent. App use was collected and ported to a secure data server at the University of California, San Francisco, which met all Health Insurance Portability and Accountability Act (HIPAA) and security requirements imposed by the university. Participants were not compensated for using the apps. Note that the eligibility criteria for randomization required that participants had (1) either an iPhone or Android smartphone, and (2) an iPad 2.0 or newer. Participants who met criteria as per requirement (1) but not (2) were given Project: EVO if they had an iPhone, or Health Tips if they had an Android.

### Outcome Assessments

The primary outcome measures were of depression (PHQ-9) and function (SDS [34]), with these scores captured weekly for the first 4 weeks of treatment, then at 8 and 12 weeks (see Multimedia Appendix 1 for discussion of other exploratory outcomes). Participants were paid US $20.00 for completing assessments at the 4-, 8-, and 12-week marks. Because all assessment was conducted using assessment software, procedures for blinding research assistants was not necessary.

### Data Analysis

All analyses were modeled on an intent-to-treat approach. We used hurdle models [35] to estimate (1) predictors of app adherence and follow-up rates, (2) which variables would predict using the apps at least once (as odds ratios, ORs) and, (3) which variables would predict the number of times the apps were used (as rate ratios, RRs). ORs and RRs used standardized *z* scores as continuous predictors to facilitate interpretations associated with the relative increase in odds or rate associated with a 1-SD change on given predictors. Predictors in hurdle models included baseline PHQ-9, SDS, GAD-7, AUDIT-C, IMPACT, age, sex, level of education, marital status, employment status, minority status, whether participants endorsed other psychiatric or psychotherapeutic treatments at baseline, and condition assignment.

We characterized participant adherence in 3 ways: none=no use at all (downloading the app did not count toward use); suboptimal=some use, but never met adherence criteria for a given week *or* only met adherence criteria for 1 of the 4 weeks; and optimal=met adherence criteria for at least 2 of the 4 weeks, such that participants exactly followed the specified instructions for app use as outlined in the informative YouTube videos. For Project: EVO, we considered three 30-minute sessions per week (or 50% of the indicated amount) to be the minimum acceptable amount of treatment with respect to assessing the feasibility of self-administering a daily cognitive intervention. We used identical procedures to predict follow-up rates, but included participants from all 3 treatment conditions.

To estimate changes in depression and disability during and after the treatment period, we tested growth curve models using multilevel modeling with continuous piecewise growth curves for each period. These models used restricted maximum likelihood with all available data to reduce missing data bias [36], and included random intercepts and random effects for time. Growth curve models are well known to be better than some other methods for estimating interindividual variability in intraindividual patterns of change, including accounting for missing data in a rigorous manner [37]. We entered baseline PHQ-9 or SDS scores as control variables along with medication use due to differences between groups in this variable at baseline. Separate models tested the impact of the app conditions on remission rates. Remission was assessed by characterizing the proportion of participants who demonstrated a reduction of 50% of their pretreatment depression and disability scores [38,39].

We conducted sensitivity analyses to evaluate whether missing data biased estimates through pattern-mixture models [40], which is considered the gold standard for RCT studies [41,42]. This approach is deemed important to test the assumption that data are missing at random for multiple imputation methods [43]. We also tested whether the amount of change in depression or disability was moderated by baseline PHQ-9, GAD-7, SDS, AUDIT-C, age, or app use through baseline variable-by-time interactions.

### Sample Size and Power

A power analysis [44] indicated that 200 participants per condition would provide 0.80 power to detect whether an active treatment condition improved by 2 points on the PHQ-9 beyond the control condition (approximately Cohen *d*=0.4), assuming a 50% dropout and an alpha level of .05.

## Results

### Participant Flow, Recruitment, and Baseline Data

**Figure 1:**
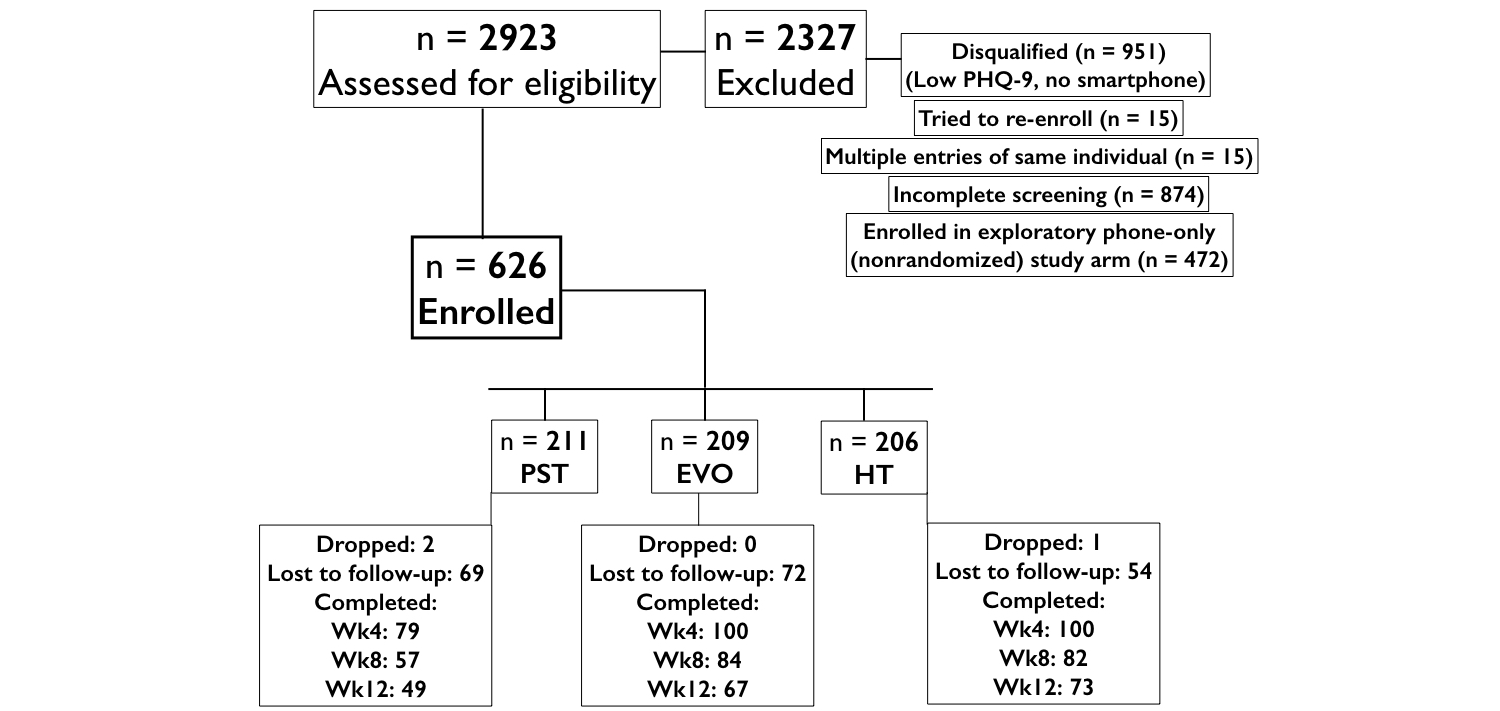
Consolidated Standards of Reporting Trials (CONSORT) diagram. EVO: Project: EVO; HT: Health Tips; PHQ-9: 9-item Patient Health Questionnaire; wk: week.

National recruitment began in August 2014, with 2923 participants screened across the 5 waves of 2-week advertising (total of 5 months of recruitment; see Consolidated Standards of Reporting Trials [CONSORT] diagram, Figure 1). A total of 626 participants had both an iPad 2.0 and a smartphone, and were randomly assigned to the 3 study arms (iPST, 211; Project: EVO, 209; Health Tips, 206). The mean age of the sample was 33.95 (SD 11.84) years, and the mean PHQ-9 score at baseline (13.64, SD 4.95) indicated the sample was moderately depressed. The proportion of individuals in our sample with a PHQ-9 total score between 5 and 10 was 23.0% (144/626), while the proportion of those with a score ≥10 was 77.0% (482/626). Only 11 participants who had a total PHQ-9 score less than 5 reported a score greater than 2 on item 10 of this tool, with their baseline total PHQ-9 being 3.09 (SD 0.83). The majority of the sample was female (494/626, 79.0%) and non-Hispanic white (376/626, 60.1%) Table 1 presents the demographic characteristics of the randomized sample, including ethnic group proportions, concurrent clinical diagnoses, and those in treatment.

**Table 1:** Sample descriptive statistics of participants using 3 different self-guided mobile apps for depression.

			EVO^a^ (n=209)		iPST^b^ (n=211)		HT^c^ (n=206)		Total (n=626)		
**Baseline variable**											
	PHQ-9^d^ score, mean (SD)		13.76	(4.9)	13.51	(5.1)	13.64	(4.9)		13.64	(4.95)
	PHQ-disability score, mean (SD)		1.34	(0.75)	1.44	(0.68)	1.40	(0.73)		1.39	(0.72)
	Age in years, mean (SD)		34.9	(12.3)	33.4	(10.9)	33.6	(12.3)		33.9	(11.84)
	Male, n (%)		51	(24.2)	48	(23.0)	33	(16.0)		132	(21.1)
	University education, n (%)		128	(60.7)	140	(67.0)	130	(63.1)		398	(63.6)
	Married, n (%)		62	(29.4)	73	(34.9)	68	(33.0)		203	(32.4)
	Employed, n (%)		144	(68.2)	156	(74.6)	141	(68.4)		441	(70.5)
**Racial/ethnic minority, n (%)**			128	(60.7)	122	(58.4)	124	(60.2)		374	(59.7)
		African American	29	(13.7)	29	(13.9)	28	(13.6)		86	(13.7)
		American Indian	4	(1.9)	1	(0.5)	1	(0)		6	(1.0)
		Asian	22	(10.4)	16	(7.7)	16	(7.8)		54	(8.6)
		White	137	(64.9)	140	(67.0)	133	(64.6)		410	(65.5)
		>1 race	19	(9.0)	21	(10.0)	26	(12.6)		66	(10.5)
		Native Hawaiian/Pacific Islander	0	(0)	2	(1.0)	2	(1.0)		4	(0.6)
		Hispanic (any race)	24	(11.4)	33	(15.8)	22	(10.6)		79	(12.6)
**First-use variable**											
	SDS^e^, mean (SD)		15.9	(7.1)	14.9	(6.7)	15.9	(7.1)		15.6	(6.96)
	GAD-7^f^, mean (SD)		10.4	(4.9)	9.2	(4.9)	10.4	(5.3)		10.0	(5.09)
	AUDIT-C^g^, mean (SD)		3.20	(2.5)	3.03	(2.2)	3.40	(2.3)		3.21	(2.38)
	Psychotic symptoms, n (%)		29	(19)	24	(20)	32	(24)		155	(21)
**Other psychiatric treatment, n (%)**			84	(58)	64	(52)	74	(56)		222	(56)
		Psychiatrist	41	(19.6)	31	(14.8)	35	(16.7)		107	(17.1)
		Therapist	41	(19.6)	32	(15.3)	30	(14.4)		103	(16.5)
		Group	15	(7.1)	10	(4.8)	5	(2.4)		30	(4.8)
		Book	26	(12.4)	23	(11.0)	35	(16.7)		84	(13.4)
		Medication^h^	63	(30.1)	43	(20.6)	39	(18.6)		145	(23.2)

^a^EVO: Project: EVO.

^b^iPST: problem-solving therapy app.

^c^HT: Health Tips.

^d^PHQ-9: 9-item Patient Health Questionnaire.

^e^SDS: Sheehan Disability Scale.

^f^GAD-7: Generalized Anxiety Disorder 7-item scale.

^g^AUDIT-C: Alcohol Use Disorders Identification Test.

^h^EVO had a significantly higher rate of baseline medication use than iPST and HT.

### Sensitivity Analysis and Moderators of Treatment Effects

The sensitivity analyses performed here to account for potential bias in the data revealed that the patterns of missing data did not predict significant differences in PHQ-9 (χ^2^_6_=8.47, *P*=.20) or SDS trajectories (χ^2^_6_=9.67, *P*=.14) during weeks 1-4. This suggests that overall levels and changes in depression during weeks 1-4 were not significantly different between those who did and did not provide follow-up data after week 4. Comparing participants who used the Project: EVO and iPST apps optimally, suboptimally, or not at all, we observed that the overall levels of depression as scored by the PHQ-9 were not different for the suboptimal or optimal groups relative to the “none” group for both Project: EVO (all *P* ≥.22) and iPST (all *P* ≥.64; see Multimedia Appendix 1). Baseline functioning, alcohol use, and age did not significantly moderate the effects of treatment condition on depression outcomes. We found that baseline depression significantly moderated changes in depression over time during weeks 4-12 for the iPST group relative to control (*P*=.02). Baseline anxiety significantly moderated changes in the SDS scores over time during weeks 4-12 for iPST (*P*=.01) but not for Project: EVO (*P*=.08) relative to control.

### Assessment Adherence

Of the participants who self-reported a PHQ-9 total score <10, 66.0% (95/144) used the survey app, compared with 64.9% (313/482) of those with a PHQ-9 score >10, with this difference in use being nonsignificant (*P*=.82). A total of 354/626 (56.6%) provided at least one follow-up assessment. Participants with at least one follow-up assessment provided an average of 5.83 (SD 2.42) of 8 possible follow-up assessments, with participants who were older (RR 1.08, *P*=.02) completing a greater number of follow-up assessments (see Table 2). Racial/ethnic minorities were more likely than nonminorities to provide at least one follow-up assessment (OR 1.26, *P*<.001; see Multimedia Appendix 1 for more on follow-up analyses, participant expectancy, and perceived study burden).

**Table 2:** Predictors of app use and follow-up completion.

					Predictors of app use													Predictors of follow-up											
					Any use					Use count					*P* value			Any follow-up					No. of follow-ups						*P* value
					OR^a^		(SE^b^)		RR^c^			(SE)					OR			(SE)		Beta			(SE)				
**Baseline variable**																													
		PHQ-9^d^		1.01		(0.12)		0.79			(0.09)		.93			0.90			(0.09)		–.23			(0.16)		.04			
		PHQ-disability		0.93		(0.11)		1.27			(0.15)		.54			0.96			(0.09)		–.17			(0.16)		.03			
		Age		1.06		(0.12)		1.15			(0.12)		.61			1.08			(0.10)		.29			(0.15)		.19			
		Male		0.89		(0.09)		0.96			(0.09)		.25			0.96			(0.08)		–.11			(0.14)		.67			
		University education		1.21		(0.13)		1.01			(0.11)		.07			1.01			(0.09)		.18			(0.14)		.92			
		Married		0.93		(0.10)		0.93			(0.09)		.53			0.84			(0.07)		–.21			(0.14)		.46			
		Employed		1.09		(0.11)		0.92			(0.09)		.42			0.97			(0.08)		–.05			(0.14)		.39			
		Racial/ethnic minority		1.04		(0.11)		0.95			(0.09)		.70			1.26			(0.11)		.25			(0.14)		.57			
		Condition: iPST^e^		1.06		(0.21)		1.11			(0.21)		.78			1.05			(0.21)		–.05			(0.33)		.60			
		Condition: EVO^f^														0.69			(0.14)		–.93			(0.33)					
**First-use variable**																													
		SDS^g^		0.97		(0.16)		1.30			(0.16)		.83			1.29			(0.24)		–.06			(0.19)		.04			
		GAD-7^h^		1.03		(0.17)		0.78			(0.10)		.86			0.76			(0.14)		–.26			(0.19)		.04			
		AUDIT-C^i^		1.03		(0.13)		0.77			(0.06)		.80			0.96			(0.14)		–.14			(0.15)		.001			
		Psychotic symptoms		1.19		(0.40)		1.02			(0.25)		.62			1.34			(0.53)		.24			(0.39)		.93			
		Other psychiatric treatment		0.77		(0.21)		1.08			(0.21)		.34			0.73			(0.23)		–.03			(0.32)		.71			

^a^OR: odds ratio.

^b^SE: standard error.

^c^RR: rate ratio.

^d^PHQ-9: 9-item Patient Health Questionnaire.

^e^iPST: problem-solving therapy app.

^f^EVO: Project: EVO.

^g^SDS: Sheehan Disability Scale.

^h^GAD-7: Generalized Anxiety Disorder 7-item scale.

^i^AUDIT-C: Alcohol Use Disorders Identification Test.

### Intervention Adherence

Because the Health Tips control did not require interaction, we report adherence for the active apps only. Among the 420 participants in the Project: EVO and iPST conditions, 243 (57.9%) did not download their assign app. Those who used their app at least once used it on average 10.78 (SD 11.44) times. Higher baseline depression (PHQ-9) and anxiety (GAD-7) were associated with less use, such that a 1-SD increase in each was associated with a 21% and 23% lower rate of adherence, respectively (RR 0.79 and 0.78, respectively, *P*=.04 and *P*=.001, respectively). However, higher disability was associated with a greater adherence (27% for PHQ-disability scale and 30% for SDS; RR 1.27 and 1.30, respectively, *P*=.047 and *P*=.04; see Table 2). None of the baseline variables were significantly associated with the likelihood of using versus not using the Project: EVO or iPST apps. Among participants with at least one use, higher baseline PHQ-9, GAD-7, and AUDIT-C scores were associated with fewer uses, while higher PHQ-disability scores and SDS scores were associated with a greater number of uses.

We further tested whether there were condition-by-baseline variable interactions that predicted use counts and the likelihood of at least one use. Marital status also interacted with treatment condition to predict having at least one instance of use (*P*=.02), such that married individuals were less likely to use iPST once (OR 0.54, *P*=.05). Baseline AUDIT-C scores significantly interacted with treatment condition to predict use counts among those with at least one use (*P*=.048). Specifically, higher AUDIT-C scores were significantly associated with lower use counts in the Project: EVO condition (RR 0.59, *P*=.001). There were no significant interactions between treatment and the variables presented in Table 2 predicting use.

**Figure 2:**
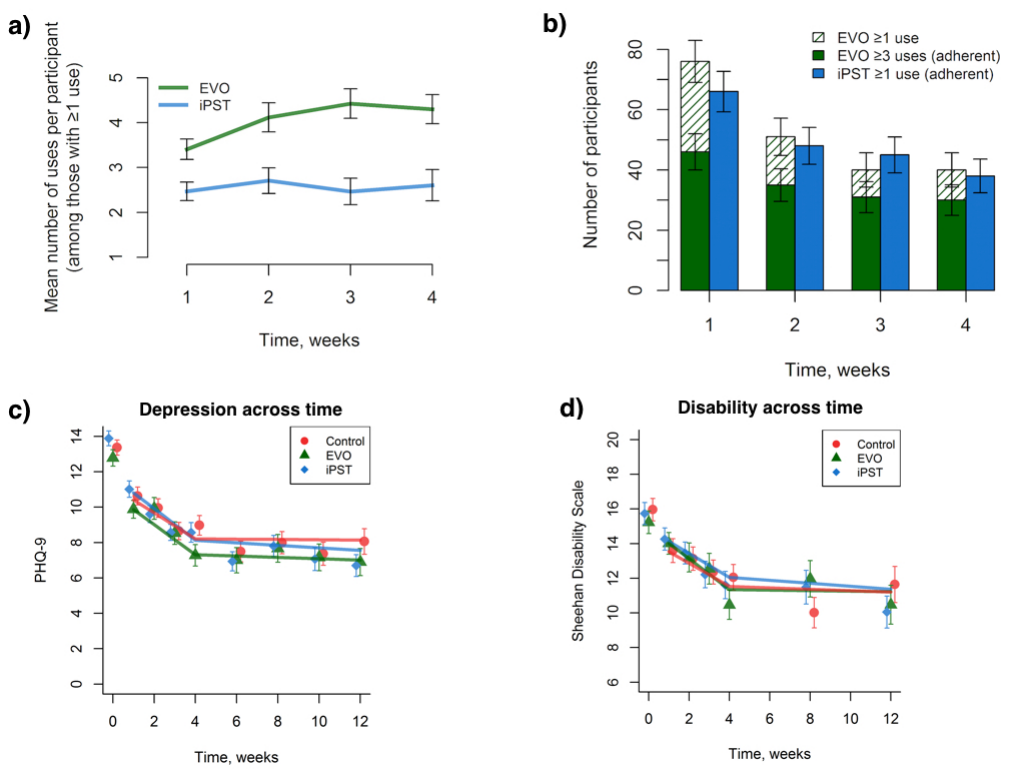
Participant intervention use and changes in primary outcome measures over time. (a) Average number of active intervention uses across the first 4 weeks of the study. (b) Number of participants using each active intervention by the level of adherence. (c) 9-item Patient Health Questionnaire (PHQ-9) depression scores over time for each intervention. (d) Sheehan Disability Scale scores over time for each intervention. Control: Health Tips; EVO: Project: EVO; iPST: problem-solving therapy app. Error bars indicate +/- 1 SE.

Baseline depression significantly interacted with treatment condition to predict the likelihood of having at least one use instance (*P*=.01), such that higher baseline depression was associated with a lower likelihood of using the Project: EVO app at least once (OR 0.73, *P*=.03; 95% CI 0.55-0.96). All app adherence significantly declined over time (log OR –0.77, SE 0.23, *z* score –3.30, *P*<.001), with no main effect of group (log OR 0.80, SE 0.51, *z* score 1.56, *P*=.12) or group-by-time interaction (log OR 0.24, SE 0.31, *z* score 0.77, *P*=.44; see Figure 2, parts a and b).

**Table 3:** Main effects of treatment on changes in depression and disability.

Fixed effects	PHQ-9^a^			SDS^b^		
	B	SE^c^	*P* value	B	SE	*P* value
(Intercept, week 4)	8.21	(0.46)	.00	11.33	(0.64)	.000
Baseline PHQ-9 or SDS	2.89	(0.22)	.00	3.91	(0.26)	.000
Medication use	1.10	(0.43)	.01	0.66	(0.53)	.21
Change per week, weeks 1-4	–0.73	(0.14)	.00	–0.67	(0.22)	.002
EVO^d^ condition	–0.52	(0.67)	.44	0.05	(0.94)	.96
iPST^e^ condition	–0.60	(0.62)	.34	0.47	(0.89)	.60
Change per week, weeks 4-12	–0.01	(0.06)	.90	–0.04	(0.10)	.69
EVO × change per week, weeks 1-4	–0.12	(0.21)	.58	–0.22	(0.33)	.50
iPST × change per week, weeks 1-4	–0.16	(0.19)	.40	–0.02	(0.31)	.94
EVO × change per week, weeks 4-12	–0.03	(0.10)	.77	0.03	(0.16)	.87
iPST × change per week, weeks 4-12	–0.06	(0.09)	.49	–0.05	(0.15)	.76

^a^PHQ-9: 9-item Patient Health Questionnaire.

^b^SDS: Sheehan Disability Scale.

^c^SE: standard error.

^d^EVO: Project: EVO.

^e^iPST: problem-solving therapy app.

### Depression Outcomes

#### Depression Symptom Severity

For the total sample, PHQ-9 scores decreased an average of 0.73 points per week during the treatment phase and did not significantly change from weeks 4-12 (see Figure 2 c). The models revealed no significant differences between Project: EVO and iPST compared with control at week 4 or week 12, and did not differ in rates of change over time (see Table 3). With respect to treatment remission (using a reduction of pretreatment scores of at least 50% as the criterion for this), 45.0% (45/100) of Project: EVO participants and 46% (36/79) of iPST participants showed improvement by the 4-week assessment, as compared with 34.0% (34/100) of the control app (χ^2^=3.36, *P*=.19).

#### Outcomes by Baseline Depression Severity

**Figure 3:**
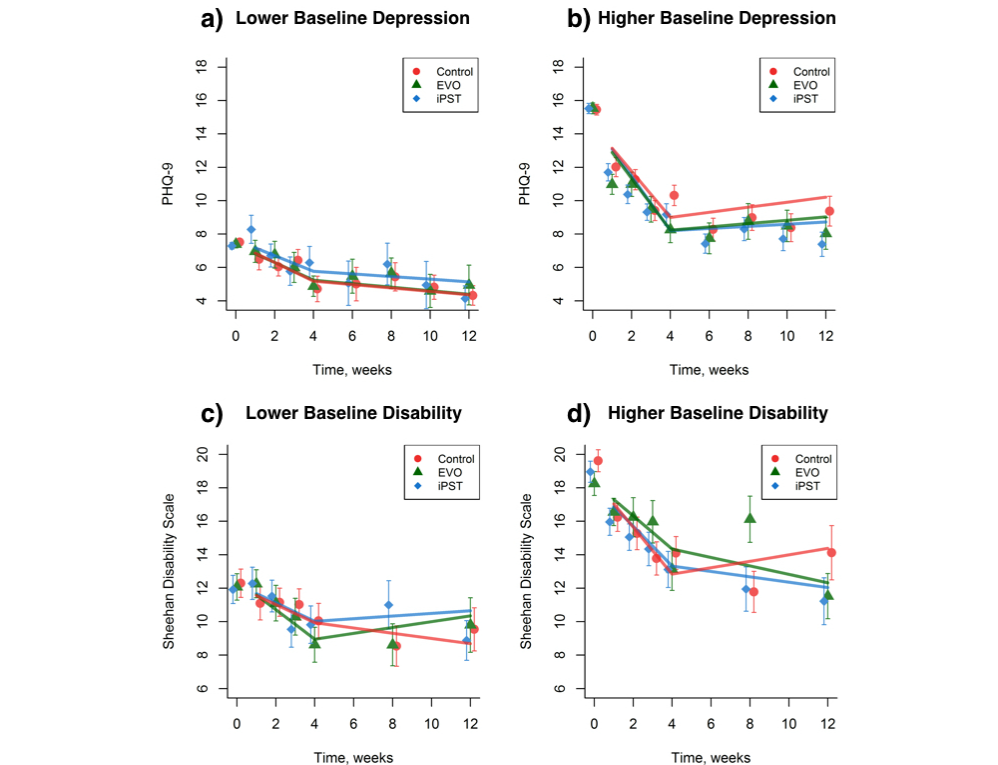
Changes in 9-item Patient Health Questionnaire (PHQ-9) and Sheehan Disability Scale scores moderated by baseline depression and by symptom severity for each intervention. (a) Individuals with lower baseline depression by group. (b) Individuals with higher baseline depression by group. (c) Individuals with lower baseline anxiety by group. (d) Individuals with higher baseline anxiety by group. Control: Health Tips; EVO: Project: EVO; iPST: problem-solving therapy app. Error bars represent +/- 1 SE.

A total of 144 participants were classified as having mild symptoms of depression (PHQ-9 ≤9) and 482 with moderate symptoms of depression at baseline (PHQ-9 ≥10). For the mildly depressed subgroup, Project: EVO and iPST did not significantly differ from the control condition at any point. For the subgroup with higher baseline depression, depression was significantly lower at week 12 for the iPST condition (difference=1.79, SE 0.76, *t*_201_=–2.36, *P*=.02) but not the Project: EVO condition (*P*=.15) relative to control (see Figure 3, parts a and b). With regard to remission, moderately depressed participants had a greater response to Project: EVO (28/56, 50%) and iPST (39/79, 49%) than to the Health Tips arm (24/76, 32%; χ^2^_2_*=* 6.46, *P*=.04). We found no difference between treatment groups at week 4 (χ^2^_2_=0.84, *P*=.66) or week 8 (χ^2^_2_=1.79, *P*=.41).

### Disability Outcomes

#### Disability Symptom Severity

Disability decreased an average of 0.67 points per week during weeks 1-4 and did not significantly change from weeks 4-12 (see Figure 2 d). The Project: EVO and iPST groups’ disability did not significantly differ from that of controls at week 4 or week 8 or in the rates of change over time (see Table 3).

#### Outcomes by Baseline Disability Severity

A total of 159 participants were classified as having mild disability (SDS ≤15) and 237 with moderate disability at baseline (SDS >15). For the both subgroups, Project: EVO and iPST did not significantly differ from the control condition at week 4 or week 8. With regard to remission, moderately disabled participants showed no difference at weeks 4 and 8 for the Project: EVO (13/42, 31%; and 7/30, 23%), iPST (20/54, 37%; and 16/45, 36%), and Health Tips conditions (15/61, 25%; and 18/44, 41%; χ^2^_2_=2.09 and 2.48, respectively, *P*=.35 and .29, respectively; see Figure 3 c). For the mildly disabled group, Project: EVO yielded higher rates of recovery at 4 weeks (18/34, 53%) compared with the Health Tips group (9/38, 24%; χ^2^_1_=5.36, *P*=.02), with similar recovery observed between the iPST (14/40, 35%) and Health Tips arms (χ^2^_1_=0.72, *P*=.40; see Figure 3 d), with no group differences observed at week 8 (χ^2^_2_=1.31, *P*=.52).

## Discussion

### Principal Findings

To our knowledge, this is the first fully remote randomized clinical field trial of mobile apps for depression in a national sample in the United States. Given the increased interest of health care organizations in the potential of mobile technology to address service gaps for mental health [45], the data from this study provide important information on the impact apps can have in depression care. Our findings suggest that apps designed to engage cognitive correlates of depression had the strongest effect on depressed mood for people with more moderate levels of depression.

As has been found in smaller-scale studies, people who download mood apps tend to use these tools as intended for no more than 2 weeks [21,46]. Despite poor adherence in app use, the benefits seem to be positive in terms of mood and functioning. While we cannot rule out that the outcomes were not simply regression to the mean for the full sample, it does appear that for those who are more significantly impaired, apps that are designed to target specific cognitive deficits implicit in depressive disorders, in this case cognitive control, are more effective than our control intervention. These findings, coupled with data from smaller proof-of-concept studies of the impact of apps on mood [17,47], suggest that for some people with mild to moderate depression, mobile apps could serve as an alternative means of treatment, particularly for those where mental health resources are scarce (eg, ethnic minorities). This was evidenced by the fact that our sample was much more representative of the US population than is typically found in mental health settings across the United States, with service utilization among ethnic/racial groups here being comparable with use rates in the United States [48]. However, these (and all) interpretations should be reviewed with caution given that, while this was an RCT, it was primarily conducted as a feasibility trial. Thus, this was not a mechanistic trial specifically designed to assess and compare the efficacy of these interventions, but rather to provide methodological insights for future work in this space.

### Interpretation and Limitations

While our findings showed a positive impact on depression and disability outcomes, half of the enrolled participants never downloaded their assigned app despite having completed eligibility screens, consent forms, and baseline assessment. This is not an uncommon phenomenon in research of this nature: for example, nearly all self-guided Internet-based studies experience dropout rates as high as 90% very early in the study timeline [49], and a recent RCT comparing a mood app versus a control app had an 82% dropout rate [50]. Although our final sample was large enough to test the effects of apps on outcomes, the findings should be interpreted cautiously. For the mildly depressed subsample (those individuals with a PHQ-9 between 5 and 10), changes in mood could be attributed to regression to the mean, as spontaneous recovery with such mild depression is not uncommon. However, it is rare for people with a score of ≥15 on the PHQ-9 to simply “get better” (ie, regress to the mean), with our findings in the moderately depressed subsample demonstrating a significant difference between interventions. Furthermore, given the variation in outcomes based on symptom severity, and recent data finding that people with PHQ-9 scores of less than 10 do not have a clinical depression [22], we do not recommend that depression outcomes studies recruit participants with such mild presentations, unless the sample is very large (>10,000).

In addition to the high early study dropout rate, most participants did not use their assigned intervention apps as instructed, a ubiquitous effect across apps even given that these apps differed in content, user experience, and other features. This pattern of use mirrors other field trials of mental health apps that report app use typically wanes over the course of 2 weeks [51]. While limited adherence restricts our ability to test the efficacy of these interventions when used as designed, our data provide useful insight into how individuals typically interact with self-guided treatment apps. Indeed, the participants in this study were relatively tech savvy compared with those less technologically inclined, and we would expect to see some differences comparing these 2 groups, especially in the likelihood of using such apps for treatment purposes. For those less technologically inclined (or even those in this study), having a personal connection with a coach or other peers may be a critical element to encourage longer-term app use. Additionally, future research should also investigate the utility of apps that dynamically shift as user goals shift. The human-computer interaction field has recently demonstrated that user needs and interest in app-supported care vary over time, and engagement with app-based care may hinge on the ability of apps to dynamically adjust to the users’ needs and interests [52].

Mobile apps still have a potential place in the treatment of mood disorders. Adherence to these tools, particularly when delivered as a self-guided treatment, is a problem, and methods for improving adherence should be explored.

## Appendix

Multimedia Appendix 1 Supplemental materials: Follow-up rates; eTable 1 (PHQ-9 by week as a function of Group Adherence for each active intervention); Adaptive Cognitive Evaluation (ACE); Cognitive Therapy (EVO); Problem Solving Therapy; Health Tips; Expectancy; Perceived Participant Burden.

Multimedia Appendix 2 CONSORT-EHEALTH checklist V1.6.1.

## Abbreviations

AUDIT-C: Alcohol Use Disorders Identification Test
CONSORT: Consolidated Standards of Reporting Trials
GAD-7: Generalized Anxiety Disorder 7-item scale
HIPAA: Health Insurance Portability and Accountability Act
IMPACT: Improving Mood-Promoting Access to Collaborative Treatment
OR: odds ratio
PHQ-9: 9-item Patient Health Questionnaire
RCT: randomized controlled trial
RR: rate ratio
SDS: Sheehan Disability Scale
SMS: short message service
