# Supplementary material for: The Use and Effectiveness of Mobile Apps for Depression: Results From a Fully Remote Clinical Trial
Source: J Med Internet Res. 2016 Dec 20;18(12):e330. doi: 10.2196/jmir.6482 (PMC5209607; doi:10.2196/jmir.6482)
Supplement: Multimedia Appendix 1 [file jmir_v18i12e330_app1.pdf]

Supplemental Materials

Follow-Up Rates 2

eTable 1. PHQ-9 by week as a function of Group Adherence for each active intervention 3

Adaptive Cognitive Evaluation (ACE) 4

Cognitive Therapy 6

Health Tips 7

Expectancy 8

Perceived Participant Burden 8

References 8

eFigure 1. Follow-up Rates Over Time, by Condition.

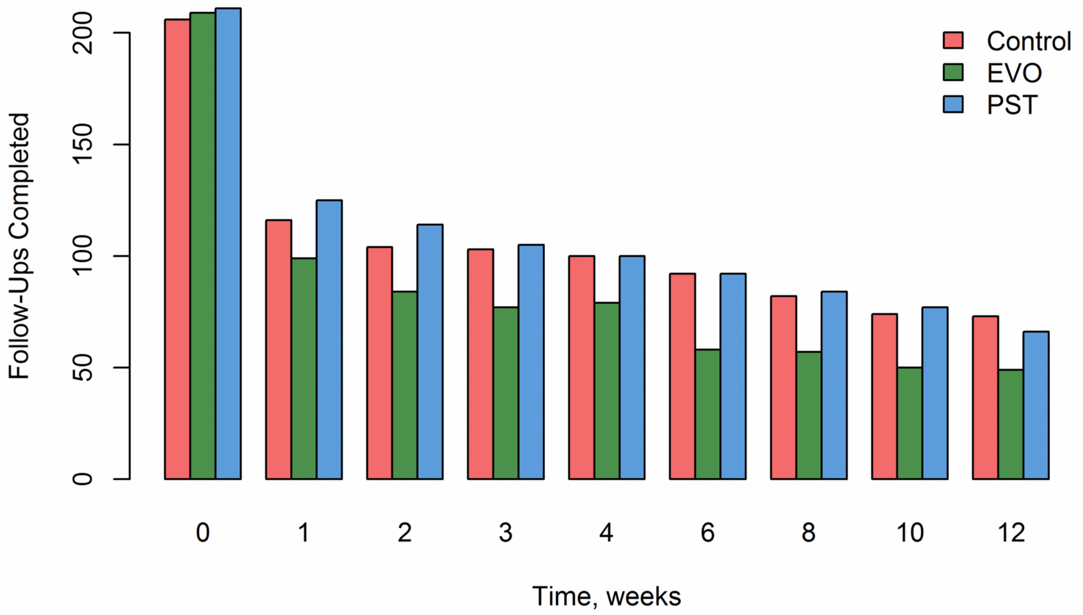

Follow-Up Rates

A generalized mixed model predicting the likelihood of providing follow-up data at each time point indicated a decreased likelihood of providing follow-up data over time for all participants (OR_{week} = 0.64, p < .001). After controlling for time, participants in the EVO condition were less likely to provide follow-up data over time relative to the control condition ($OR_{EVO \times week}$ = 0.70, *p* = .02); there was no difference in follow-up rates over time for the PST condition ($OR_{PST \times week}$ = 0.85, *p* = .17).

There was a significant treatment-by-baseline AUDIT interactions that predicted the number of follow-ups completed (p = .02). Specifically, higher AUDIT scores were associated with fewer follow-ups completed in the EVO condition (B = -0.64, p = .03) and non-significantly related to fewer follow-ups in the Educational control (B = -0.44, p = .07), but were unrelated to follow-ups in the PST condition (B = .35, p = .14). There was a significant treatment-by-employment status interaction predicting the likelihood of having at least one follow-up (p = .01) and predicting the number of follow-ups completed (p = .03). In subgroup analyses, being employed was associated with a greater number of follow-ups (B = 0.56, p = .02) and a greater likelihood of having at least one follow-up (OR = 1.42, p = .02) for the EVO condition but was unrelated to follow-up in the PST or control conditions.

eTable 1. PHQ-9 by week as a function of Group Adherence for each active intervention

iPS	WEEK	ADHERENCE GROUP	PHQ-9	EVO	WEEK	ADHERENCE GROUP	PHQ-9
	0	none	13.4		0	none	14.2
	1	none	11.0		1	none	9.8
	2	none	9.7		2	none	11.3
	3	none	7.9		3	none	8.6
	4	none	9.1		4	none	7.2
	6	none	7.6		6	none	5.5
	8	none	7.5		8	none	7.5
	10	none	6.0		10	none	8.2
	12	none	6.8		12	none	6.4
	0	optimal	13.8		0	optimal	11.4
	1	optimal	10.5		1	optimal	9.3
	2	optimal	9.4		2	optimal	9.1
	3	optimal	9.0		3	optimal	7.8
	4	optimal	8.2		4	optimal	6.7
	6	optimal	6.9		6	optimal	6.8
	8	optimal	8.1		8	optimal	7.1
	10	optimal	7.7		10	optimal	6.9
	12	optimal	6.8		12	optimal	6.8
	0	suboptimal	14.8		0	suboptimal	14.0
	1	suboptimal	12		1	suboptimal	10.7
	2	suboptimal	10.0		2	suboptimal	10.1
	3	suboptimal	8.6		3	suboptimal	9.9
	4	suboptimal	9.1		4	suboptimal	8.5
	6	suboptimal	6.2		6	suboptimal	8.6
	8	suboptimal	7.3		8	suboptimal	9.4
	10	suboptimal	6.2		10	suboptimal	7.6
	12	suboptimal	5.2		12	suboptimal	7.8

Adaptive Cognitive Evaluation (ACE)

First described in Anguera et al., (2016; BMJ Innovations)

ACE was meant to be used before participants used their study-specific app, and then completed again at the week 4, week 8, and week 12 time points in the study to monitor potential changes in cognitive function. However, a very few number of participants actually completed their assessments at the 8 or 12 week marks, thus we did not examine data from these time points and focused exclusively on the differences at baseline and the 4 week mark. We also selected one task from each cognitive control domain (attention, working memory, goal management) that had the most participants in total who completed these tasks. These tasks comprised the STROOP task, the Spatial Span task (a derivative of the Corsi block task), and the task switch paradigm. Findings from these tasks are presented below:

eTable 2. Performance on ACE cognitive control tasks

	Intervention X Session Effect	Session Main Effect	EVO Session effect* (pre N= 71, post N=53)	iPST Session Effect* (pre N=75, post N=37)	Health Tips Session Effect* (pre N=30, post N=8)
Attention (Stroop)					
COST: RT Variance	F=1.50, p=0.23	F=7.7, p=.006	P=.002	P=.50	P=.1
COST: Mean RT	F=.40, p=0.67	F=6.4, p=.012	P=.001	P=.033	P=.57
			(pre N= 73, post N=51)	(pre N=64, post N=33)	(pre N=26, post N=10)
Working Memory (Spatial Span)					
Overall Count	F=.44, p=.64	F=4.9, p=.03	P=.25	P=.25	P=.12
			(pre N= 65, post N=49)	(pre N=34, post N=6)	(pre N=62, post N=41)
Goal Management (Task Switch)					
SWITCH: Mean RT	F=1.2, p=.30	F=.54, p=.46	P=.88	P=.19	P=.345
SWITCH: RT Variance	F=4.5, p=.014	F=40.2, p<.0001	P<.0001	P=.114	P<.0001

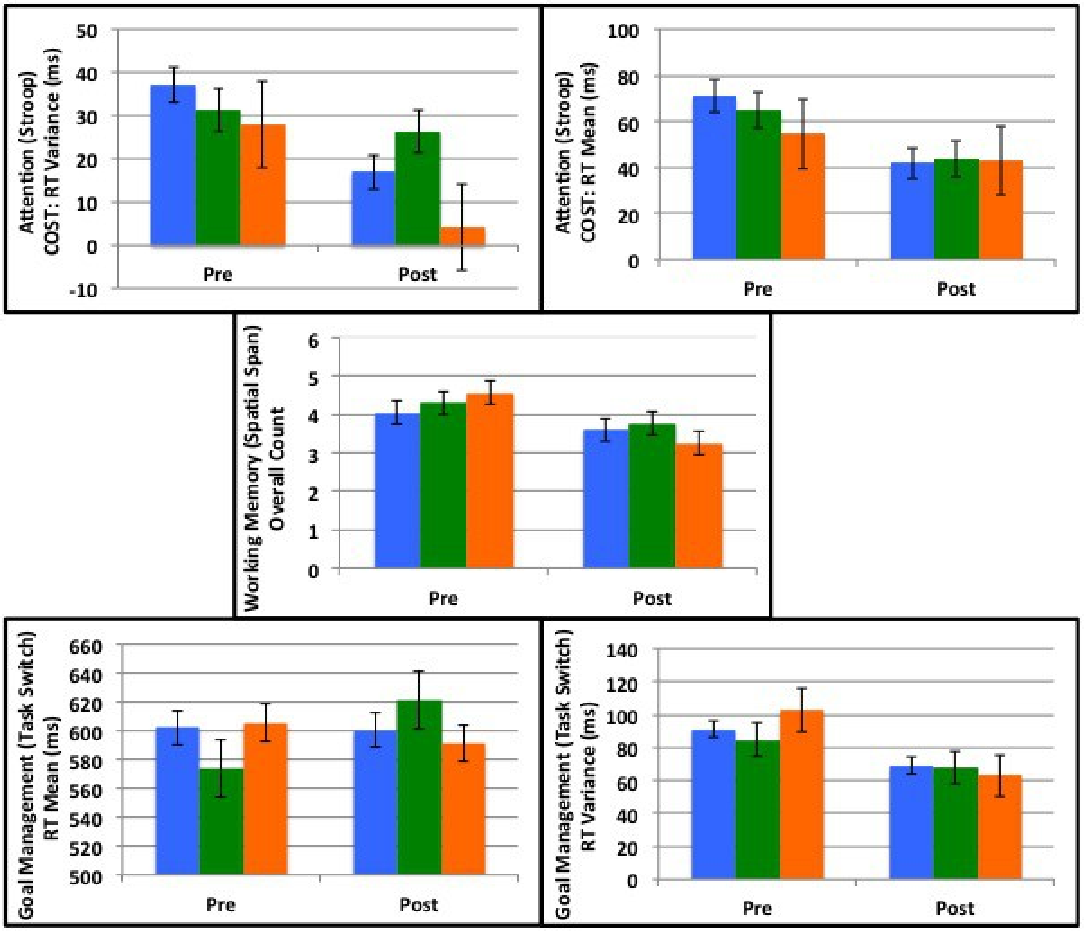

eFigure3. Performance on ACE cognitive control tasks by group

Cognitive Therapy

First described in Anguera et al., (2016; BMJ Innovations)

Project: EVO™ is a mobile immersive video game designed to modulate cognitive functioning based upon a related cognitive training intervention, NeuroRacer[1].

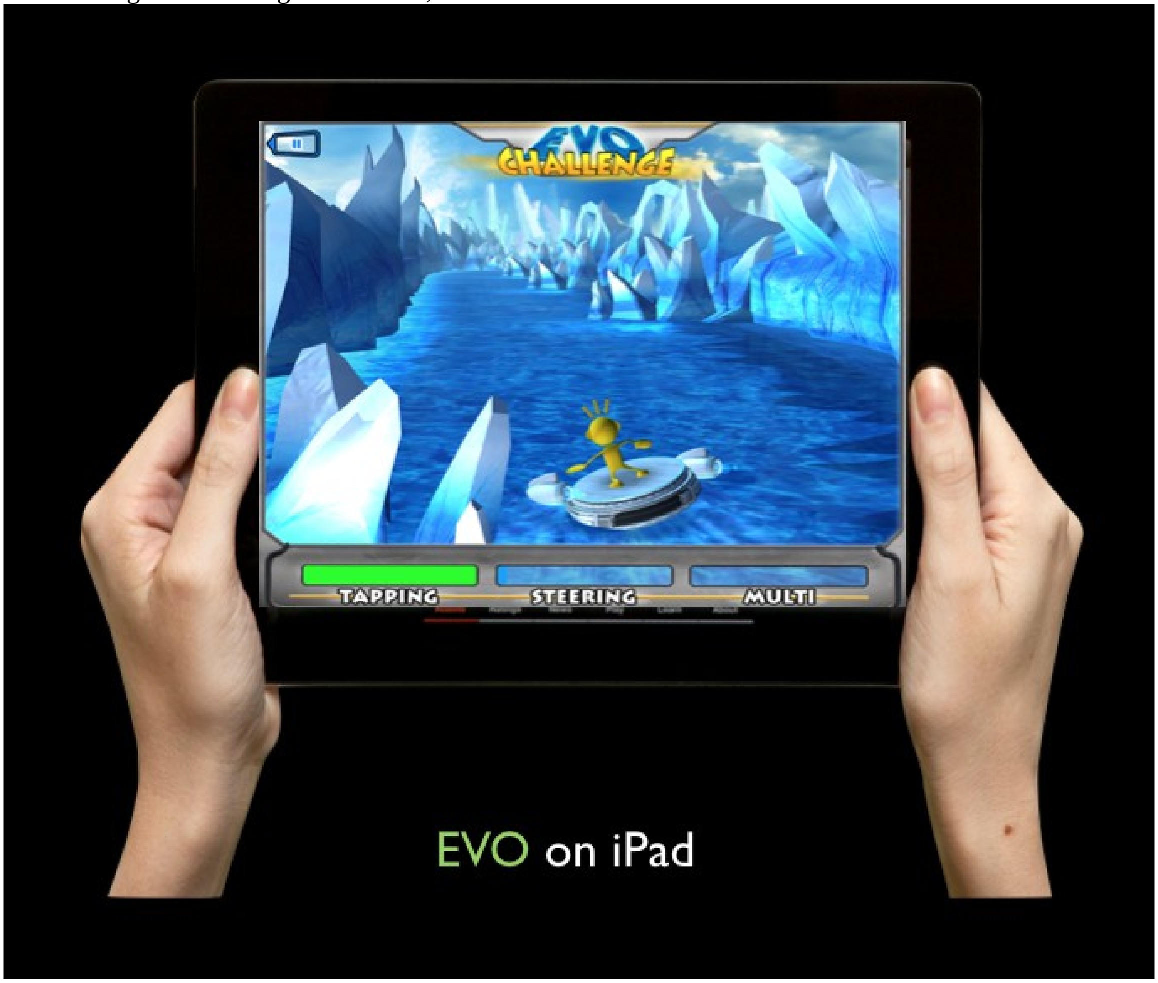

eFigure 5. Project: EVO cognitive training platform. Images from during game play

Problem Solving Therapy

First described in Anguera et al., (2016; BMJ Innovations)

iProblemSolve is based on social problem-solving therapy developed by Nezu and D'Zurilla [2], focuses on using a systematic, rational problem-solving approach to improve functioning.

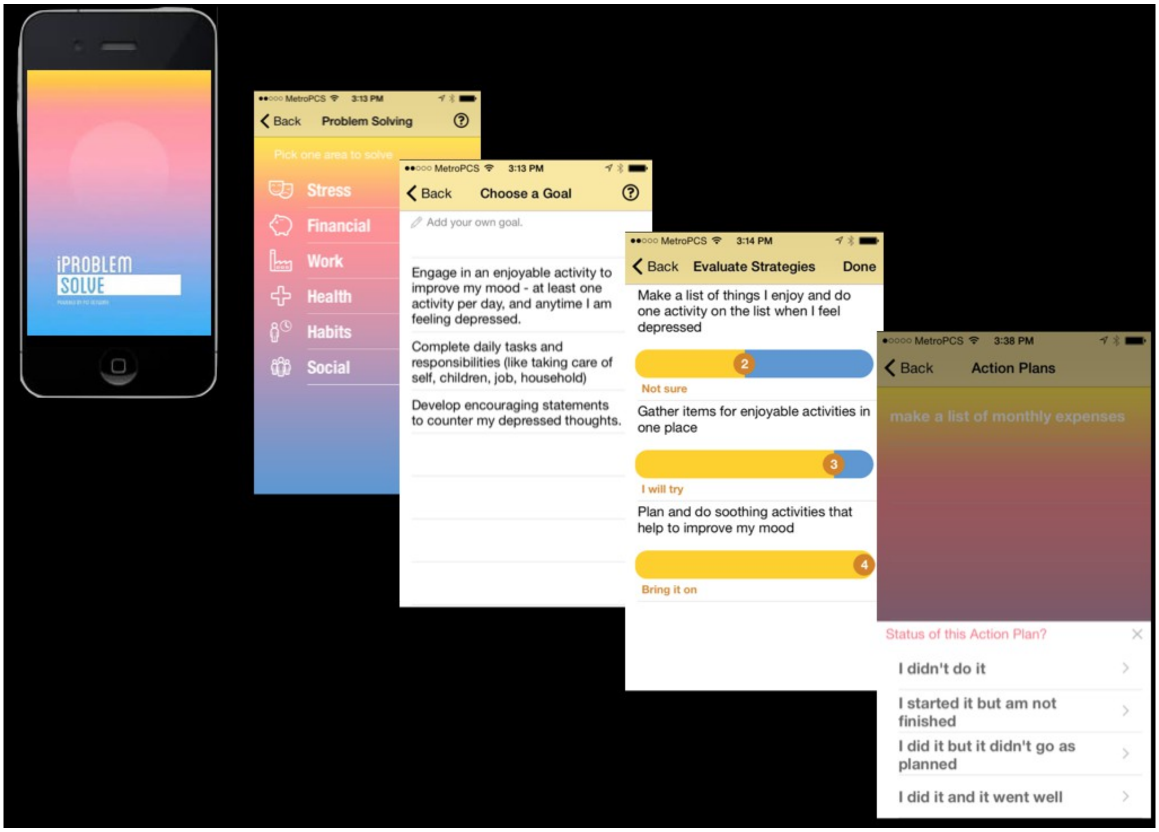

eFigure 5. iProblemSolve application. iPS application images showing common steps experienced.

Health Tips

First described in Anguera et al., (2016; BMJ Innovations)

Through the Ginger.io platform, Health Tips were delivered daily with suggestions for overcoming depressed mood at the beginning of each day. Examples of suggestions include self-care (e.g., going outside for sunlight, taking a shower), physical and social activities (e.g., speaking with loved ones, going to an event). Note that while daily advice was provided, these tips act in a similar fashion to supportive-control treatments as they are not tied to any specific theory. Furthermore, participants were not required to act on the health tip.

Expectancy

After completing the first day of their intervention as well as the cognitive assessment, participants were sent brief surveys that specifically asked about their expectancy following the prescribed use of their study-specific application on their mental health, cognitive abilities, and performance on everyday tasks. In addition, participants were also asked about their beliefs in the efficacy of various treatments on cognition and/or mental health (e.g., psychological treatments, self-improvement programs, medical care, and cognitive enhancement), as well as their experience with playing video games.

Of participants that engaged in their study app, 122 participants in the primary arm completed an expectancy survey. The expectancy surveys indicated no difference amongst participants across each of the primary arms of the study with respect to anticipated improvements in overall functioning ($F[2, 120] = 1.78$, $p = .17$) or depressive symptoms ($F[2, 120] = 1.00$, $p = .37$). In terms of the secondary arms, 106 individuals responded to the expectancy surveys. Health Tips users believed their application would have more of an impact on their mood than EVO users, $t(99.97) = 2.54$, $p = .01$, but there were no differences between groups in anticipated improvements in overall functioning, $t(104.26) = 1.56$, $p = .12$. There was no difference amongst those receiving outside treatment with respect to anticipated overall ($t(182.72) = .64$, $p = .52$) or mental health benefits ($t(173.97) = .28$, $p = .78$). Finally, there was no correlation between PHQ-9 score and the anticipated benefits for overall functioning ($r = -.02$, $p = .77$) or psychological function ($r = .08$, $p = .25$).

Perceived Participant Burden

An exit survey was sent to all participants who had shown some level of activity upon completion of the study. Of the 725 surveyed, 170 (23%) responded. Only 37 (22%) had dropped out of the study. There were 35 individuals (21%) from the PST/iPad group; 23 (14%) from the EVO iPad group; 37 (22%) from the Health Tips/iPad group; 33 (19%) from the EVO iPhone group, and 42 (25%) from the Health Tips Android group. Participants were asked on a scale of 1 (low) to 10 (high) of a burden it was to be in the BRIGHTEN Study. The median score was 2.5, indicating low overall burden.

A one-way ANOVA examining participants' feelings about study burden indicated a differential amount of burden dependent upon on the intervention assigned, ($F(2, 167) = 8.17$, $p < .001$). Bonferroni-corrected pairwise comparisons revealed that participants in the EVO conditions experienced more participant burden than participants in the PST ($t(77.73) = 2.64$, $p = .01$, Cohen's $d = .56$) or Health Tips conditions ($t(102.96) = 3.78$, $p < .001$, Cohen's $d = .69$). Unsurprisingly, individuals that believed they were not being paid enough for their time were significantly more likely to drop out, $x_1^2 = 9.22$, p < .01, OR = 3.19. Overall, however, all intervention arms believed they were being paid enough for their time ($x_2^2 = 1.07$, p = .59). The intervention arms nearly differed in terms of perceived intervention effectiveness, $x_2^2 = 5.64$, p = .06, with EVO trending toward lower perceived effectiveness.

References

1. Anguera JA, Boccanfuso J, Rintoul JL, et al. Video game training enhances cognitive control in older adults. *Nature.* 2013;501(7465):97-101.
2. Nezu AM. Efficacy of a social problem-solving therapy approach for unipolar depression. *Journal of consulting and clinical psychology.* 1986;54(2):196-202.
